# Arts, culture and sports engagement and self-rated health: a cross-sectional population-based study in southern Sweden

**DOI:** 10.1186/s12889-024-20031-9

**Published:** 2024-09-28

**Authors:** Martin Lindström, Mirnabi Pirouzifard, Anita Jensen

**Affiliations:** Social Medicine and Health Policy, Department of Clinical Sciences and Center for Primary Health Care Research, Lund University and Region Skåne, Malmö, 205 02 Sweden

**Keywords:** Arts participation, Theatre/cinema attendance, Arts exhibition attendance, Sports event attendance, Social participation, Health-related behaviours, Self-rated health (SRH), Sweden

## Abstract

**Background:**

International research demonstrates an association between arts and culture activities and health and wellbeing. A similar association exists for sports event attendance and health. The aim of this study was to investigate associations between arts and culture engagement and attending sports events during the past year and self-rated health (SRH).

**Methods:**

A cross-sectional study. A public health survey with three reminders was sent to a stratified random sample of the adult 18–84 population in Scania in the southernmost part of Sweden in October-December 2019. The weighted response rate was 44%, and 40,087 total respondents were included in the present study. Analyses were performed in logistic regression models with multiple adjustments for age, education, country of birth, leisure-time physical activity (LTPA), smoking, alcohol consumption, and economic stress.

**Results:**

The prevalence of poor SRH was 32.8% among women and 27.6% among men. The prevalence of theatre/cinema attendance was 63.2% among women and 55.2% among men, arts exhibition/museum attendance was 41.0% among women and 36.0% among men, and for sports attendance it was 33.8% among women and 48.2% among men. All items included in the final models showed statistically significant associations with self-rated health in bivariate logistic regression models. In the multiple model, adjusted for all covariates including both men and women, the odds ratios (ORs) of poor SRH were statistically significant OR 1.21 (95% confidence interval: 1.14–1.29) for not visiting theatre/cinema during the past year, OR 1.11 (1.04–1.17) for not visiting arts exhibition and OR 1.31 (1.24–1.39) for not visiting a sports event.

**Conclusions:**

Significant associations between arts and culture engagement and sports event attendance, and SRH were observed, although effect measures were comparatively low for arts and cultural engagement. The results may be useful for informing public health promotion and prevention strategies.

**Supplementary Information:**

The online version contains supplementary material available at 10.1186/s12889-024-20031-9.

## Background

Numerous population studies have shown associations between self-rated health (SRH) and attending arts and culture activities. These studies have reported associations between participating in arts and culture activities and higher self-rated satisfaction with life and general happiness [1]; positive life-satisfaction and self-esteem in adolescents [2]; positive association with SRH and quality of life (QOL) [3]; improved SRH for women and reduction of all-cause mortality for men [4]; and reduced risk of incident or persistent depression [5]. As well as other health and wellbeing related outcomes, including mortality [6], cancer mortality [7] eudaimonic wellbeing [8] wellbeing and less social loneliness [9]. Additionally, individuals with a higher frequency of arts and culture engagement are also more likely to report good SRH [10]. SRH is regarded as a crucial metric in epidemiological research, serving as a reliable predictor for both mortality and morbidity [11]. Consequently, it holds significant value as an indicator of coping resources and can influence health-related behaviours that ultimately impact outcomes [12]. Considering global health as contextual – social, cultural and behavioural and the growing number of people facing challenges with mental and physical health—it is imperative to adopt novel and interdisciplinary strategies for both prevention and the healthcare system [13]. This necessitates interdisciplinary and cross-sector collaborations [14]. The global adoption of arts for health promotion and illness prevention has seen a rise [15, 16], also in the Nordic region [17]. Arts and culture activities have the potential to significantly enhance social participation by fostering a sense of community, inclusion, and overall wellbeing [16]. However, in terms of health promotion, it is important to consider that not ‘one size fits all’, and it is therefore imperative to promote different activities for health and wellbeing.

Arts and culture activities have in previous studies been associated with sociodemographic, chronic disease and health-related behaviour factors [18]. Furthermore, SRH has in previous studies been associated with sociodemographic, health-related behaviour and economic stress factors [19] and attending sports events has been shown to have positive associations with some aspects of subjective wellbeing (i.e. life satisfaction and a sense of life being worthwhile) as well as loneliness [20]. This suggests that attending arts and sports events may offer platforms for public health promotion. Given that both arts and culture attendance and sport event attendance are associated with positive health and wellbeing outcomes, we wanted to compare the SRH outcomes of exhibition/museum and theatre/cinema attendance with sports event attendance to investigate any differences in their associations with SRH, respectively. The aim of this study was to investigate associations during the past year between theatre/cinema attendance, arts exhibition/museum attendance and SRH, adjusting for age, sex, education, country of birth, leisure-time physical activity, smoking, alcohol consumption, economic stress, and sports event attendance. In addition, we compared the SRH outcomes of theatre/cinema and arts exhibition attendance with sports event attendance in the adult population. To our knowledge, this is the first study to make such a comparison.

## Methods

### Study population

In October–November 2019, a cross-sectional public health survey was conducted in Scania, the southernmost part of Sweden, by the regional public authority *Region Skåne* that is responsible for the healthcare system in the region. A stratified random sample of 107,300 persons of the population of Scania aged 18–84 years old was approached with a survey in Swedish (paper and digital format) and English (digital). Two reminder letters were posted to initial non-responders in paper and digitally. The survey questionnaire contained 69 items concerning health, health-related behaviours, healthcare, demographics, social and economic conditions, social relations, social support, and social capital. Three reminders were sent including two postal letters with the second letter also containing the survey on paper. The third reminder was sent to the two youngest age groups, 18–29 years old and 30–44 years old, as a text message (sms) due to the lower response rates among these age groups. A total of 42,564 respondents answered the questionnaire. The weighted response rate was 44% and the unweighted response rate 40%. All analyses in Tables 1, 2, and 3 and Table S1 are weighted using a population weight derived from the stratification of the random sample since the 30 city parts of the four major cities and the 29 smaller municipalities vary in population size and in composition of age, sex, country of birth and education. The objective was to display the public health situation also in smaller geographic areas and strata. The application of the population weight in the analysis meant that larger geographic areas and strata, where a smaller proportion of the population was sampled, regained their original weight in the calculations to achieve prevalence and other epidemiological effect measures representative of the entire population. It should be noted that weighted statistical analyses including logistic regression models produce results very close to the unweighted results. The present study includes 40,087 respondents (17,876 men and 22,211 women) for reasons of internally missing answers on one or several items included in the study (2477 internally missing in total). *Statistics Sweden* created the stratified sample as well as the population weight that compensates for the stratification according to age, sex and geographic units (municipalities/city parts).

**Table 1:** Descriptive characteristics (prevalence) (%) of the 2019 public health survey. The 2019 public health survey in Scania. Total population *n* = 40,087. Weighted prevalence

	All (*n* = 40,087)	Women (*n* = 22,211)	Men (*n* = 17,876)	*p*-value
Self-rated health				< 0.001
Poor	30.2	32.8	27.6	
Good	69.8	67.2	72.4	
Theatre/cinema during the past year				< 0.001
No	40.8	36.8	44.8	
Yes	59.2	63.2	55.2	
Arts exhibition/museum during past year				< 0.001
No	61.5	59.0	64.0	
Yes	38.5	41.0	36.0	
Sports event during the past year				< 0.001
No	59.0	66.2	51.8	
Yes	41.0	33.8	48.2	
Age				< 0.001
Age 18–34	29.0	28.5	29.4	
Age 35–44	17.0	16.6	17.4	
Age 45–54	17.2	17.3	17.2	
Age 55–64	14.9	14.77	14.9	
Age 65–84	21.9	22.8	21.1	
Education				< 0.001
Primary school	19.1	16.9	21.3	
Secondary school (gymnasium)	41.8	40.3	43.3	
Higher than secondary school	39.1	42.8	35.4	
Country of birth				< 0.001
Sweden	75.0	75.8	74.3	
Other Nordic countries	2.6	2.7	2.5	
Europe, other than Nordic countries	10.8	10.8	10.9	
Born outside Europe	11.6	10.7	12.3	
Intensive LTPA				< 0.001
0 min/week	25.7	26.1	25.2	
Less than 30 munites/week	18.9	19.4	18.4	
30–59 min/week	15.2	16.4	14.0	
60–89 min/week	11.3	11.8	10.8	
90–119 min/week	8.0	8.3	7.7	
2 h or more	20.9	18.0	23.9	
Moderate LTPA				< 0.001
0 minuter/week	4.5	4.0	5.0	
Less than 30 min/week	10.9	9.9	12.0	
30–59 min/week	17.9	17.8	17.9	
60–89 min/week	16.0	15.9	16.2	
90–149 min/week	16.0	16.3	15.6	
150–299 min/week	16.8	17.9	15.8	
5 h or more	17.9	18.2	17.5	
Smoking				< 0.001
Yes, daily smoker	8.9	8.8	8.9	
Yes, but not daily smoker	6.1	5.4	6.9	
No, non-smoker	85.0	85.8	84.2	
Alcohol consumption				< 0.001
4 times/week or more	6.7	4.8	8.5	
2–3 times/week	22.0	19.4	24.7	
2–4 times/month	30.2	29.6	30.8	
1 time/month or less	24.1	26.9	21.3	
Never	17.0	19.3	14.7	
Economic stress				< 0.001
No	87.8	87.3	88.4	
Economic stress once during the past year	6.0	6.2	5.8	
Economic stress several times during the past year	6.2	6.5	5.8	

*p*-value: Pearson Chi Square test, 2-sided

**Table 2:** Odds ratios with 95% confidence intervals (ORs with 95% CIs) of poor self-rated health (SRH) for theatre cinema during the past year, arts exhibition/museum during the past year, sports event during the past year, sex, education, country of birth, intensive leisure-time physical activity (LTPA), moderate leisure-time physical activity (LTPA), smoking, alcohol consumption and economic stress. All analyses are conducted in *bivariate* unadjusted logistic regression models. The 2019 public health survey in Scania. Total population *n* = 40,087. Weighted

		**Crude model**		
	**Reference category**		**95% CI**	
Theatre/cinema during the past year	Yes	1.94***	1.85	2.04
Arts exhibition/museum during the past year	Yes	1.68***	1.60	1.76
Sports event during the past year	Yes	2.07***	1.96	2.17
Sex	Men	1.28***	1.22	1.34
Age 18–34	Age 65–84	0.43***	0.40	0.46
Age 35–44		0.49***	0.45	0.53
Age 45–54		0.61***	0.56	0.65
Age 55–64		0.81***	0.76	0.87
Education: Primary school	Higher than secondary school	1.94***	1.82	2.08
Education: Secondary school		1.56***	1.48	1.64
Nordic countries other than Sweden	Sweden	1.17*	1.00	1.36
Europe, other than Nordic countries		1.03	0.94	1.11
Born outside Europe		1.06	0.97	1.15
Intensive LTPA 0 min/week	2 h/ week or more	3.84***	3.56	4.13
Intensive LTPA less than 30 min/week		3.00***	2.77	3.25
Intensive LTPA 30–59 min/week		2.06***	1.89	2.25
Intensive LTPA 60–89 min/week		1.66***	1.51	1.83
Intensive LTPA 90–119 min/week		1.41***	1.27	1.56
Moderate LTPA 0 min/week	5 h/ week or more	3.41***	2.99	3.87
Moderate LTPA less than 30 min/week		2.33***	2.13	2.54
Moderate LTPA 30–59 min/week		1.65***	1.53	1.78
Moderate LTPA 60–89 min/week		1.29***	1.19	1.39
Moderate LTPA 90–149 min/week		1.20***	1.10	1.30
Moderate LTPA 150–299 min/week		1.06	0.98	1.15
Yes, daily smoker	No, non-smoker	2.06***	1.90	2.24
Yes, but not daily smoker		1.06	0.96	1.17
Alcohol consumption 4 times/week or more	Never	0.73***	0.65	0.81
Alcohol consumption 2–3 times/week		0.49**	0.45	0.53
Alcohol consumption 2–4 times/month		0.51***	0.48	0.55
Alcohol consumption 1 time/month or less		0.78***	0.73	0.84
Economic stress once during the past year	No	1.75***	1.57	1.94
Economic stress several times past year		3.83***	3.48	4.21

Significance levels: * *p* < 0.05, ** *p* < 0.01, *** *p* < 0.001. Weighted Odds Ratios (ORs) with 95% confidence intervals (95% CIs). Bootstrap method (1000 replicates) for variation estimation

**Table 3:** Odds ratios with 95% confidence intervals (ORs with 95% CIs) of poor self-rated health (SRH) according to visit to theatre/cinema, arts exhibition/museum and sports event during the past year. Adjusted for sex, age, education, country of birth, intensive leisure-time physical activity (LTPA), moderate leisure-time physical activity (LTPA), smoking, alcohol consumption and economic stress in multiple logistic regression models 0–4. The 2019 public health survey in Scania. Men and women collapsed (All) and stratified (men and women). Total population *n* = 40,087. Weighted

		**Model 0**			**Model 1**			**Model 2**			**Model 3**			**Model 4**		
	**Ref**		**95% CI**		**OR**	**95% CI**		**OR**	**95% CI**		**OR**	**95% CI**		**OR**	**95% CI**	
**All**																
Theatre/cinema during the past year	Yes	1.55***	1.47	1.63	1.44***	1.37	1.52	1.40***	1.32	1.48	1.24***	1.17	1.31	1.21***	1.14	1.29
Arts exhibition/museum during the past year	Yes	1.30***	1.24	1.37	1.37***	1.29	1.44	1.27***	1.20	1.34	1.11***	1.05	1.18	1.11***	1.04	1.17
Sports event during the past year	Yes	1.80***	1.71	1.90	1.67***	1.58	1.76	1.67***	1.58	1.77	1.35***	1.27	1.43	1.31***	1.24	1.39
**Men** **(*n*** **= 17,876)**																
Theatre/cinema during the past year	Yes	1.64***	1.51	1.78	1.45***	1.34	1.58	1.40***	1.29	1.52	1.28***	1.17	1.39	1.26***	1.16	1.38
Arts exhibition/museum during the past year	Yes	1.22***	1.12	1.32	1.27***	1.17	1.38	1.17***	1.08	1.28	1.05	0.96	1.15	1.04	0.95	1.14
Sports event during the past year	Yes	1.71***	1.58	1.84	1.68***	1.55	1.81	1.69***	1.56	1.82	1.36***	1.25	1.47	1.32***	1.21	1.43
**Women (*n* = 22,211)**																
Theatre/cinema during the past year	Yes	1.54***	1.44	1.65	1.43***	1.34	1.54	1.39***	1.29	1.49	1.20***	1.11	1.30	1.17***	1.08	1.26
Arts exhibition/museum during the past year	Yes	1.40***	1.31	1.50	1.45***	1.36	1.55	1.35***	1.26	1.45	1.16***	1.08	1.25	1.16***	1.08	1.25
Sports event during the past year	Yes	1.76***	1.64	1.89	1.67***	1.55	1.79	1.67***	1.55	1.79	1.35***	1.25	1.46	1.32***	1.22	1.42

Model 0: Unadjusted; Model 1 adjusted for sex and age; Model 2 additionally adjusted for education and country of birth; Model 3 additionally adjusted for intensive leisure-time physical activity (LTPA), moderate leisure-time physical activity (LTPA), smoking and alcohol consumption; Model 4 additionally adjusted for economic stress; Significance level: *** *p* < 0.001. Weighted Odds Ratios (ORs) with 95% confidence intervals (95% CIs). Bootstrap method (1000 replicates) for variation estimation

### Dependent variable

Self-rated health (SRH) was assessed with the question “What is your general state of health?” with the alternatives “Very good”, “Good”, “Neither good nor poor”, “Poor” and “Very poor”. This item was dichotomised with the two first alternatives as “Good” and the three latter as “Poor”.

### Independent variables

*Theatre or cinema visit during the past year* was assessed with the question “Have you visited the theatre or cinema during the past year”. *Arts exhibition or museum visit during the past year* was assessed similarly. These items are part of a social participation item whether or not the respondent had taken part in formal groups, informal groups and activities during the past year. The 13 activities included study circle/course at work, other study circle/course, union meeting, other meeting, theatre/cinema, arts exhibition, church/religious service attendance, sports event, letter to editor of newspaper/journal, demonstration, night club/entertainment, big gathering of relatives and private party. A 14th alternative was none of the above.

*Sports event during the past year* was assessed with the question “Have you attended a sports event during the past year?” from the same item as theatre/cinema and arts exhibition/museum (see above).

Men and women were collapsed in Tables 2 and 3. In supplementary Table SI men and women were both collapsed and analysed separately. *Age* was analysed as a continuous variable in Table 3. *Education* was defined into higher than secondary school, upper secondary school and primary school. *Country of birth* was categorised into Sweden, other Nordic countries, the rest of Europe and those born outside Europe. Sex, age, education and country of birth were derived from register-based data from Statistics Sweden. *Leisure-time physical activity* (LTPA) during one week was defined in two ways following the 2018 International criteria for physical activity. Intensive physical activity (affecting breathing intensity including e.g., running, gymnastics, strength training, ball sport) had alternatives 0 min/no time, less than 30 min, 30–59 min, 60–89 min, 90–119 min and 2 h or more. Every day (moderate) physical activity contained alternatives 0 min/no time, less than 30 min, 30–59 min, 60–89 min, 90–149 min, 150–299 min, and 5 h or more. S*moking* was assessed with the question “Do you smoke?”, and was divided into non-smoker, intermittent (non-daily) smoker and daily smoker. *Alcohol consumption* during the past twelve months was assessed with the item “How often have you consumed alcohol during the past 12 months?” and included the alternatives never, once per month or less, 2–4 times/month, 2–3 times/week, and 4 times/week or more. *Economic stress* was assessed with the question “Have you during the past 12 months had difficulties paying for food, rent, bills etc.?”, and concerned the number of times the respondent had experienced difficulties paying food, rent, bills during the past 12 months including alternatives “Never”, “Yes, on one occasion”, and “Yes, on several occasions”. Sex, age, education, country of birth, LTPA (intensive and daily moderate), smoking, alcohol consumption and economic stress were analysed as confounders.

### Statistics

Prevalence (%) of all variables are presented stratified by sex using chi-square tests for all categorical variables (Table 1). Crude odds ratios (ORs) with 95% confidence intervals (95% CIs) of associations between each separate independent variable and poor SRH were calculated in *bivariate* logistic regression models (Table 2). Odds ratios (ORs) with 95% confidence intervals (95% CIs) of poor SRH according to theatre/cinema visit during the past year, arts exhibition/museum visit during the past year, and sports event visit during the past year were calculated using multiple logistic regression models unadjusted for other independent variables (model 0). The following models were adjusted for age and sex (model 1), additionally adjusted for education and country of birth (model 2), additionally adjusted for intensive and moderate LTPA, smoking and alcohol consumption during the past year (model 3), and finally additionally adjusted for economic stress (model 4). The analyses including models 0–4 in Table 3 were conducted both with men and women collapsed and stratified by sex (Table 3). Sampling variability can be investigated without assumptions concerning the distribution of the study population using bootstrap analysis with 1000 iterations. Odds ratios (ORs) with 95% confidence intervals of no visit to theatre/cinema, no visit to arts exhibition and no visit to a sports event during the past year were calculated with men, age group 65–84, higher than secondary school, born in Sweden, intensive LTPA 2 h/week or more, moderate LTPA 5 h/week or more, non-smoking, never alcohol consumption and no economic stress during the past year as references (Supplementary Table S1). The SAS software 9.4 was used for calculations [21].

## Results

The prevalence of poor SRH was significantly higher among women (32.8%) than among men (27.6%). Women had visited theatre/cinema (63.2%) and arts/exhibition/museum (41.0%) to a higher extent than men (55.2% and 36.0%, respectively). In contrast, the prevalence of having visited a sports event during the past year was higher among men (48.2%) than among women (33.8%). Men were significantly younger, less educated (in terms of length of education) and born abroad, particularly outside Europe, than women. The prevalence of LTPA, smoking, alcohol consumption and economic stress are also displayed in Table 1.

Table 2 displays that all independent variables were significantly associated with the dependent dichotomised variable SRH in *bivariate* unadjusted logistic regression analyses. Nevertheless, only those born in other Nordic countries had a significantly higher OR of poor SRH, OR 1.17 (1.00–1.36), compared to the category born in Sweden. No visit to theatre/cinema, no visit to arts exhibition/museum, no visit to sports event, being a woman, higher age and lower level of formal education was associated with significantly higher ORs of poor SRH. Daily smoking and lower levels of intensive and moderate LTPA showed significantly higher ORs of poor SRH. Alcohol consumption during the past year showed significantly lower ORs of poor SRH than no consumption during the past year. Economic stress was significantly associated with higher ORs of poor SRH (Table 2).

The multiple logistic regression analyses in Table 3 show that the respondent group that had not visited theatre/cinema during the past year had significantly higher ORs of poor SRH throughout models 0–4. In model 1, adjusting for only sex and age, an OR 1.44 (1.37–1.52) was observed. In model 4, it had decreased to OR 1.21 (1.14–1.29). Similar patterns were observed for the respondent group that had not visited arts exhibition/museum during the past year. The OR 1.37 (1.29–1.33) of poor SRH in model 1 was lowered to OR 1.11 (1.04–1.17) in model 4 and still statistically significant. The respondent group that had not visited a sports event during the past year displayed an OR 1.67 (1.58–1.76) of poor SRH in model 1 and an OR 1.31 (1.24–1.39) in model 4. The ORs of poor SRH for not having visited theatre/cinema, arts exhibition/museum and sports event were particularly lowered between models 2 and 3 when the health-related behaviours were introduced in the model. Similar patterns were observed in the analyses including models 0–4 stratified by sex, with the exception that the OR of poor SRH for arts exhibition/museum attendance among men became not statistically significant in model 3 after health-related behaviours were added, OR 1.05 (0.96–1.15). This pattern remained for visits to arts exhibition/museum among men in model 4, OR 1.04 (0.95–1.14).

Supplementary Table S1 shows that all covariates/confounders were significantly associated with no visit to theatre/cinema, arts exhibition/museum, and sports event during the past year. Women displayed significantly lower ORs of no visit to theatre/cinema and arts exhibition/museum compared to men, but significantly higher ORs of no visit to sport event compared to men. Young respondents displayed significantly lower ORs of no visit to theatre/cinema, arts exhibition/museum, and sports event than the 65–84 years age group. Groups with low education, born abroad, low intensive LTPA, low moderate LTPA, daily smoking and economic stress displayed significantly higher ORs of no visit to theatre/cinema, arts exhibition and sports event during the past year than their respective reference groups (high education, born in Sweden, high intensive and moderate LTPA, no smoking and no economic stress, respectively). Alcohol consumption displayed lower ORs of no visit to all three activities compared to the no alcohol consumption reference group (Table S1).

## Discussion

In this study, we have found that statistically significant associations between arts and culture engagement and SRH remained throughout the multiple logistic regression models after adjustments for relevant items including sports event attendance. We also found statistically significant associations between sports event attendance and SRH. The study suggests that the variables (arts and culture engagement and sports event attendance) are factors that may contribute to good SRH. These findings are in line with results from our previous study which showed a significant association between arts, culture activity and lower mortality [18]. However, it also illustrates a noticeable difference between the sexes, namely that women had visited theatre/cinema (63.2%) and arts/exhibition/museum (41.0%) to a higher extent than men (55.2%, 36.0%, respectively) and, in contrast, that the prevalence of having attended a sports event during the past year was higher among men (48.2%) than women (33.8%). As such, it indicates that attending sports events is more popular among men whereas arts and culture engagement is higher among women, which may be useful knowledge in terms of promoting customised health-promoting activities.

Better reported SRH among participants who visit theatre/cinema, arts exhibition/museum and sports event compared to the group that does not participate in these activities is partly, although not completely, explained by a healthier lifestyle. Health-related behaviours such as high levels of LTPA and being a non-smoker are often combined with cultural engagement and visits to sports event in the population. The results imply that an active and healthy lifestyle includes both traditional health-related behaviours and a wider array of health-promoting cultural and sports event activities.

However, reverse direction of causality, i.e., bidirectional associations such as poor health during the past year, may prevent attendance in arts and culture activities or sports events. Individuals with better SRH may be more likely to engage in LPTA, and conversely, engaging in LTPA can also improve SRH outcomes independent of these covariates. In addition, poor health can impact an individual's SES through decreased earning potential.

Research suggests that watching sports induces both positive and negative emotions [16], which in turn may cause intense physical and psychological reactions [22, 23]. Similar findings are associated with engagement in the arts, where research suggests that arts engagement can create prosocial behaviours [24] including psychological wellbeing, physiological and positive normative outcomes (including civic engagement) [25].

Furthermore, sports event attendance suggests group emotions (collectively felt emotions irrespective of individual affiliation) which provide an opportunity for social interaction. Sports event attendance can also build group identity and belonging which in turn can alleviate loneliness and boost levels of wellbeing [20]. In terms of visits to the performance arts events, research put forward that connecting with others, sharing emotions that emerge during a performance, and belonging to a specific social world were motivating factors for visits [26]. Also, social connections formed through the arts can protect against loneliness [27].

On a societal level, both arts and culture engagement and attending sports activities can be platforms of social participation for preserving or strengthening communities, creating a sense of place and social connectedness [16, 18, 28, 29]. On an individual level, engagement in creative activities and attending sports events can create multiple opportunities for learning, personal growth, and improving communication with others which in turn can have positive health, wellbeing, and quality of life effects [5, 11, 20, 22–25].

### Strengths and limitations

This study is the first, to our knowledge, to examine the associations between participation in arts and culture activities, attending sports events and SRH during the past year, and to compare these associations with associations between sports events attendance and SRH in the same models (Table 3). The study is large, and population-based. It should be noted that the effect measures of particularly the associations between theatre/cinema and arts exhibition/museum attendance and SRH are comparatively low and only statistically significant because the population sample is large, which is a reason to be cautious when interpreting the results. The weighted response rate was 44% and the unweighted response rate 40%. This response rate is similar to most other population-based public health surveys in Sweden and other high-income countries around the year 2019. Men, young people and people with lower levels of formal education were somewhat underrepresented, but the risk of important selection bias is still low to moderate for two reasons. First, well known associations between confounders/covariates and SRH are as expected from previous findings in the international literature both regarding strength of associations and directions of associations (see Table 2). Second, the underrepresentation of men, young people and people with low education is reasonably limited. The stratified sampling procedure considered geography (municipality, city parts), sex and age.

SRH with five alternative answers is a validated and well-established item prospectively associated with mortality. This five-alternative item is mostly dichotomised as “good” and “poor” in the same way as in our study [30]. The smoking item is valid [31]. The two moderate and intensive LTPA items follow the 2018 international physical activity guidelines [32]. The items visit to theatre/cinema, arts exhibition, and sports event at least once during the past year are parts of the social participation index which has been used in Sweden since the 1970s. The validity of these items is thus closely related to the validity of the social participation index variable which has high validity and reproducibility [33, 34], see also [35]. Still, the items do not measure the intensity of these activities, i.e., how many times during the past 12 months the respondent participated in each activity. Confounders were associated with poor SRH (Table 2) and associated with the independent variables visit to theatre/cinema, arts exhibition/museum, and sport event during the past year (Supplementary Table S1), and confounders were adjusted for in the multiple logistic regression models in Table 3. Other health variables than SRH were not included in the analyses because variables such as e.g., self-assessed chronic disease and GHQ12 are fully or partially entailed in the SRH item. The multiple logistic regression analyses assessing statistical associations between arts and culture participation and SHR were adjusted for relevant confounders and covariates including sports event attendance, age, sex, education, country of birth, LTPA, smoking, alcohol consumption and economic stress. The cross-sectional study design prohibits causal inference and survey participation rates are decreasing in Sweden as well as in many other countries.

Future studies regarding the association between arts and culture engagement and SRH should include a longitudinal study design following a study cohort with at least three observation points over time, i.e. a full panel data study design [36].

## Conclusions

This study found that both visits to theatre/cinema and visits to arts exhibition/museum during the past year remained significantly associated with good SRH in the multiple logistic regression analyses when adjusted for relevant covariates, although the effect measures were small. Women were more likely to engage in arts and culture activities, visits to theatre/cinema and visits to arts exhibition/museum while men more frequently attended sports events. These findings suggest that both types of activities contribute to good SRH, and highlight gender differences in preferences, which could inform tailored public health promotion and prevention strategies and policies.

## Supplementary Information

Supplementary Material 1.

## Data Availability

The regional public authority Region Skåne is the owner and administrator of data related to public health data used in this study. For more information on how to access archived data see: Statistik och analys—Utveckling Skåne (skane.se).

## Abbreviations

AoP: Arts on Prescription
GHQ12: General Health Questionnaire 12
LTPA: Leisure-time physical activity
ORS: Outcome Rating Scale
SRH: Self-rated health

## Data availability

No datasets were generated or analysed during the current study.
