# Supplementary material for: Arts, culture and sports engagement and self-rated health: a cross-sectional population-based study in southern Sweden
Source: BMC Public Health. 2024 Sep 28;24:2654. doi: 10.1186/s12889-024-20031-9 (PMC11437888; doi:10.1186/s12889-024-20031-9)
Supplement: Supplementary file 1 — Supplementary Material 1. [file 12889_2024_20031_MOESM1_ESM.docx]

| **Table S1.** **Odds ratios with 95% confidence intervals (ORs with 95% CIs) of no visit to theatre/cinema, arts exhibition/museum and sports event during the past year in bivariate unadjusted logistic regression models. The 2019 public health survey in Scania. Men and women combined. Total population n=40087. Weighted.** | | | | | | | | | | |
| --- | --- | --- | --- | --- | --- | --- | --- | --- | --- | --- |
|  |  | No theatre/cinema during the past year | | | No arts exhibition/museum during the past year | | | No sports event during the past year | | |
|  | **Ref** | **OR** | **95% CI** | | **OR** | **95% CI** | | **OR** | **95% CI** | |
| Sex | Men | **0.72***** | 0.69 | 0.75 | **0.81***** | 0.77 | 0.84 | **1.83***** | 1.75 | 1.91 |
| Age 18-34 | Age 65-84 | **0.38***** | 0.36 | 0.40 | **0.90**** | 0.85 | 0.96 | **0.52***** | 0.49 | 0.55 |
| Age 35-44 |  | **0.53***** | 0.49 | 0.57 | **0.82***** | 0.80 | 0.88 | **0.45***** | 0.42 | 0.49 |
| Age 45-54 |  | **0.54***** | 0.50 | 0.58 | **0.82***** | 0.77 | 0.88 | **0.42***** | 0.39 | 0.45 |
| Age 55-64 |  | **0.77***** | 0.72 | 0.83 | 0.96 | 0.89 | 1.03 | **0.65***** | 0.60 | 0.69 |
| Education: Primary school | Higher than secondary school | **3.52***** | 3.30 | 3.76 | **4.07***** | 3.78 | 4.38 | **1.86***** | 1.74 | 1.99 |
| Education: Secondary school |  | **2.12***** | 2.02 | 2.23 | **3.01***** | 2.86 | 3.17 | **1.21***** | 1.15 | 1.26 |
| Nordic countries other than Sweden | Sweden | **1.52***** | 1.33 | 1.73 | 1.01 | 0.87 | 1.15 | **1.62***** | 1.40 | 1.88 |
| Europe, other than Nordic countries |  | **1.74***** | 1.61 | 1.88 | **1.31***** | 1.20 | 1.42 | **1.79***** | 1.64 | 1.95 |
| Born outside Europe |  | **2.84***** | 2.62 | 3.09 | **2.09***** | 1.91 | 2.28 | **2.42***** | 2.20 | 2.67 |
| Intensive LTPA 0 minutes/week | 2 hours/ week or more | **2.91***** | 2.72 | 3.11 | **2.02***** | 1.89 | 2.15 | **5.04***** | 4.70 | 5.40 |
| Intensive LTPA less than 30 minutes/week |  | **2.02***** | 1.88 | 2.16 | **1.40***** | 1.31 | 1.50 | **3.21***** | 2.98 | 3.44 |
| Intensive LTPA 30-59 minutes/week |  | **1.49***** | 1.38 | 1.61 | **1.11**** | 1.03 | 1.19 | **2.40***** | 2.23 | 2.59 |
| Intensive LTPA 60-89 minutes/week |  | **1.17***** | 1.08 | 1.27 | 0.93 | 0.86 | 1.00 | **1.59***** | 1.47 | 1.71 |
| Intensive LTPA 90-119 minutes/week |  | 0.95 | 0.86 | 1.05 | **0.81***** | 0.74 | 0.88 | **1.32***** | 1.20 | 1.44 |
| Moderate LTPA 0 minutes/week | 5 hours/ week or more | **3.64***** | 3.21 | 4.14 | **6.56***** | 5.48 | 7.86 | **4.32***** | 3.71 | 5.02 |
| Moderate LTPA less than 30 minutes/week |  | **2.28***** | 2.09 | 2.48 | **3.16***** | 2.87 | 3.47 | **2.33***** | 2.13 | 2.54 |
| Moderate LTPA 30-59 minutes/week |  | **1.48***** | 1.38 | 1.60 | **1.97***** | 1.83 | 2.12 | **1.56***** | 1.45 | 1.67 |
| Moderate LTPA 60-89 minutes/week |  | 1.06 | 0.98 | 1.14 | **1.33***** | 1.23 | 1.43 | **1.18***** | 1.10 | 1.26 |
| Moderate LTPA 90-149 minutes/week |  | **0.85***** | 0.79 | 0.92 | 1.03 | 0.96 | 1.10 | 1.07 | 1.00 | 1.15 |
| Moderate LTPA 150-299 minutes/week |  | **0.81***** | 0.75 | 0.87 | **0.84***** | 0.78 | 0.90 | 1.02 | 0.95 | 1.09 |
| Yes, daily smoker | No, non-smoker | **2.18***** | 2.00 | 2.36 | **2.35***** | 2.15 | 2.58 | **1.94***** | 1.77 | 2.12 |
| Yes, but not daily smoker |  | 0.95 | 0.86 | 1.05 | **1.16**** | 1.05 | 1.27 | 1.02 | 0.92 | 1.13 |
| Alcohol consumption 4 times/week or more | Never | **0.37***** | 0.33 | 0.41 | **0.26***** | 0.23 | 0.29 | **0.43***** | 0.39 | 0.48 |
| Alcohol consumption 2-3 times/week |  | **0.25**** | 0.23 | 0.27 | **0.25***** | 0.23 | 0.27 | **0.28***** | 0.26 | 0.30 |
| Alcohol consumption 2-4 times/month |  | **0.26***** | 024 | 0.28 | **0.33***** | 0.30 | 0.35 | **0.29***** | 0.27 | 0.32 |
| Alcohol consumption 1 time/month or less |  | **0.43***** | 0.40 | 0.46 | **0.60***** | 0.55 | 0.65 | **0.52***** | 0.48 | 0.57 |
| Economic stress once during the past year | No | **1.32***** | 1.19 | 1.46 | **1.41***** | 1.27 | 1.56 | **1.44***** | 1.29 | 1.60 |
| Economic stress several times past year |  | **1.79***** | 1.63 | 1.97 | **1.85***** | 1.66 | 2.05 | **1.99***** | 1.80 | 2.20 |
| Significance levels: * p<0.05, ** p<0.01, *** p<0.001. Weighted Odds Ratios (ORs) with 95% confidence intervals (95% CIs). Bootstrap method (1000 replicates) for variation estimation. | | | | | | | | | | |
